# Does online dating lead to higher sexual risk behaviour? A cross-sectional study among MSM in Amsterdam, the Netherlands

**DOI:** 10.1186/s12879-016-1637-5

**Published:** 2016-06-14

**Authors:** Titia Heijman, Ineke Stolte, Ronald Geskus, Amy Matser, Udi Davidovich, Maria Xiridou, Maarten Schim van der Loeff

**Affiliations:** Department of Infectious Diseases, Public Health Service of Amsterdam, PO box 2200, 1000 CE Amsterdam, The Netherlands; Center for Infection and Immunology Amsterdam (CINIMA), Academic Medical Center (AMC), Amsterdam, The Netherlands; Department of Clinical Epidemiology, Biostatistics and Bioinformatics, Academic Medical Center (AMC), Amsterdam, The Netherlands; Julius Center for Health Sciences & Primary Care, University Medical Center Utrecht (UMCU), Utrecht, The Netherlands; National Institute of Public Health and the Environment, Centre for Infectious Disease Control (RIVM), Bilthoven, The Netherlands

**Keywords:** Men who have sex with men, Casual sex partners, Condom use, HIV, Unprotected anal intercourse, Online dating

## Abstract

**Background:**

Men having sex with men (MSM) frequently use the Internet to find sex partners. We examined the association between unprotected anal intercourse (UAI) with partners dated online and with partners dated offline (met elsewhere), and examined whether differences can be explained by self-perceived HIV status of the index and knowledge of partnership characteristics.

**Methods:**

MSM were recruited at the Sexually Transmitted Infections Clinic in Amsterdam, in 2008–2009. Participants completed a questionnaire concerning sexual behaviour. Only men reporting both online and offline casual sex partners were included. We assessed the association between online/offline partner dating and UAI, using random-effects logistic regression analysis.

**Results:**

Five hundred seventy-seven men (351 HIV-negative, 153 HIV-positive, and 73 HIV-unaware) reported UAI in 26 % of 878 online, and 23 % of 903 offline casual partnerships. The crude OR of online dating for UAI was 1.36 (95 % CI 1.03–1.81). HIV-positive men were more likely to report UAI than HIV-negative men (49 % vs. 28 % of partnerships). Adjusted for demographic characteristics, online dating had no significant effect on UAI among HIV-negative and HIV status-unaware men, but HIV-positive men were more likely to have UAI with online partners (aOR = 1.65 [95 % CI 1.05–2.57]). After correction for partner and partnership characteristics the effect of online/offline dating on UAI among HIV-positive MSM was reduced and no longer significant.

**Conclusions:**

Online dating was not significantly associated with UAI among HIV-negative MSM. HIV-positive MSM were more likely to practise UAI with partners dated online; however, after correction for partner and partnership characteristics, online partnership acquisition was not associated with a significantly increased risk of UAI.

**Electronic supplementary material:**

The online version of this article (doi:10.1186/s12879-016-1637-5) contains supplementary material, which is available to authorized users.

## Background

Men who have sex with men (MSM) frequently use the Internet to find sex partners. Several studies have shown that MSM are more likely to engage in unprotected anal intercourse with sex partners they meet through the Internet (online) than with partners they meet at social venues (offline) [1–3]. This implies that men who acquire partners online may be at a higher risk for sexually transmitted infections (STI) and HIV [4–6]. Although higher rates of UAI are reported with online partners, the risk of HIV transmission also depends on accurate knowledge of one’s own and the sex partners’ HIV status [7–10].

A meta-analysis in 2006 found limited evidence that acquiring a sex partner online increases the risk of unprotected anal intercourse (UAI) [3]. Many previous studies compared men with online partners to men with offline partners. However, men preferring online dating might differ in various unmeasured respects from men preferring offline dating, resulting in incomparable behavioural profiles. A more recent meta-analysis included several studies examining MSM with both online and offline acquired sex partners and found evidence for an association between UAI and online partners, which would suggest a mediating effect of more information on partners, (including perceived HIV status) on UAI [13].

With increased familiarity in sexual partnerships, for example by concordant ethnicity, age, lifestyle, HIV status, and increasing sex frequency, the odds for UAI increase as well [14–16]. We compared the occurrence of UAI in online acquired casual partnerships to that in offline acquired casual partnerships among MSM who reported both online and offline casual partners in the preceding six months. We hypothesised that MSM who date sex partners both online and offline, report more UAI with the casual partners they date online, and that this effect is partly explained through better knowledge of partner characteristics, including HIV status.

## Methods

### Setting and participants

We used data from a cross-sectional study focusing on spread of STI via sexual networks [15]. Between July 2008 and August 2009 MSM were recruited from the STI outpatient clinic of the Public Health Service of Amsterdam, the Netherlands. Men were eligible for participation if they reported sexual contact with men during the six months preceding the STI consultation, they were at least 18 years old, and could understand written Dutch or English. Individuals could participate more than once, if subsequent visits to the clinic were related to a possible new STI episode. Participants were routinely screened for STI/HIV according to the standard procedures of the STI outpatient clinic [15, 17]. The study was approved by the medical ethics committee of the Academic Medical Center of Amsterdam (MEC 07/181), and written informed consent was obtained from each participant. Included in this analysis were men who reported sexual contact with at least one casual partner dated online as well one casual partner dated offline.

### Questionnaire and variables

Participants completed a standardised anonymous questionnaire during their visit to the STI outpatient clinic while waiting for preliminary test results after their consultation with a nurse or physician. The questionnaire elicited information on socio-demographics and HIV status of the participant, the three most recent partners in the preceding six months, and information on sexual behaviour with those partners. A detailed description of the study design and the questionnaire is provided elsewhere [15, 18]. Our main determinant of interest, dating location (e.g., the name of a bar, park, club, or the name of a website) was obtained for every partner, and categorised into online (websites), and offline (physical sites) dating locations. To simplify the terminology of distinguishing the partners per dating location, we refer to them as online or offline partners.

HIV status of the participant was obtained by asking the question ‘Do you know whether you are HIV infected?’, with five answer options: (1) I am certainly not HIV-infected; (2) I think that I am not HIV-infected; (3) I do not know; (4) I think I may be HIV-infected; (5) I know for sure that I am HIV-infected. We categorised this into HIV-negative (1,2), unknown (3), and HIV-positive (4,5) status. The questionnaire enquired about the HIV status of each sex partner with the question: ‘Do you know whether this partner is HIV-infected?’ with similar answer options as above. Perceived concordance in HIV status within partnerships was categorised as; (1) concordant; (2) discordant; (3) unknown. The last category represents all partnerships where the participant did not know his own status, or the status of his partner, or both. In this study the HIV status of the participant is self-reported and self-perceived. The HIV status of the sexual partner is as perceived by the participant.

In order to explore possible disclosure of HIV status we also asked the participant whether the casual sex partner knew the HIV status of the participant, with the answer options: (1) no, (2) possibly, (3) yes. Sexual behaviour with each partner was dichotomised as: (1) no anal intercourse or only protected anal intercourse, and (2) unprotected anal intercourse. To determine the subculture, we asked whether the participant characterised himself or his partners as belonging to one or more of the following subcultures/lifestyles: casual, formal, alternative, drag, leather, military, sports, trendy, punk/skinhead, rubber/lycra, gothic, bear, jeans, skater, or, if none of these characteristics were applicable, other. Concordant lifestyle was categorised as: (1) concordant; (2) discordant. Casual partner type was categorised by the participants into (1) known traceable and (2) anonymous partners.

### Statistical analysis

We compared characteristics of participants by self-reported HIV status (using *χ*2-tests for dichotomous and categorical variables and using rank sum test for continuous variables). We compared characteristics of participants, partners, and partnership sexual behaviour by online or offline partnership, and calculated P values based on logistic regression with robust standard errors, accounting for correlated data. Continuous variables (i.e., age, number of sex partners) are reported as medians with an interquartile range (IQR), and were categorised for inclusion in multivariate models. Random effects logistic regression models were used to examine the association between dating location (online versus offline) and UAI. Likelihood ratio tests were used to assess the significance of a variable in a model.

Prior to the analyses we developed a directed acyclic graph (DAG) representing a causal model of UAI. In this model some variables were putative causes (self-reported HIV status; online partner acquisition), others were considered as confounders (participants’ age, participants’ ethnicity, and no. of male sex partners in preceding 6 months), and some were assumed to be on the causal pathway between the main exposure of interest and outcome (age difference between participant and partner; ethnic concordance; concordance in life styles; HIV concordance; partnership type; sex frequency within partnership; group sex with partner; sex-related substance use in partnership).

In order to examine the possible mediating effect of more information on partners (including perceived HIV status) on UAI, we developed three multivariable models. In model 1, we adjusted the association between online/offline dating location and UAI for characteristics of the participant: age, ethnicity, number of sex partners in the preceding 6 months, and self-perceived HIV status. In model 2 we added the partnership characteristics (age difference, ethnic concordance, lifestyle concordance, and HIV concordance). In model 3, we adjusted additionally for partnership sexual risk behaviour (i.e., sex-related drug use and sex frequency) and partnership type (i.e., casual or anonymous). As we assumed a differential effect of dating location for HIV-positive, HIV-negative and HIV status unknown MSM, an interaction between HIV status of the participant and dating location was included in all three models by making a new six-category variable. For clarity, the effects of online/offline dating on UAI are also presented separately for HIV-negative, HIV-positive, and HIV-unaware men. We performed a sensitivity analysis restricted to partnerships in which only one sexual contact occurred. Statistical significance was defined as *P* < 0.05. No adjustments for multiple comparisons were made, in order not to miss potentially important associations. As a rather large number of statistical tests were done and reported, this approach does lead to an increased risk of one or more false-positive associations. Analyses were done using the statistical programme STATA, version 13 (STATA Intercooled, College Station, TX, USA).

## Results

### Study participants and partnerships

Of the 3050 MSM who participated in the study, 2119 men reported at least one casual sex partner in the previous 6 months. In total, they reported 5278 casual sex partners. The current analysis was restricted to men who reported at least one online casual sex partner and at least one offline casual partner; this concerned 577 men with 1781 casual partners: 878 online partners and 903 offline partners.

### Characteristics of the participants

Characteristics of the participants, stratified by HIV status, are shown in Table 1. The majority of men (60.8 %) considered themselves HIV-negative, 153 men (26.5 %) HIV-positive, and 73 (12.7 %) did not know their HIV status at time of enrolment. The overall median age was 37 (IQR 30–43) years. HIV-positive men were significantly older and reported more partners than HIV-negative or HIV-unaware men (*P* < 0.001). Most participants (73.8 %) were Dutch.

**Table 1:** Characteristics of 577 men who have sex with men, stratified by self-perceived HIV status, Amsterdam, 2008-9

	Total	HIV-negative	HIV-positive	HIV unaware	*P* ^*c*^
	N(%)^a^	n(%)^a^	n(%)^a^	n(%)^a^	
N	577	351 (60.8 %)	153 (26.5 %)	73 (12.7 %)	
Demographics					
Median age in years (IQR)	37 (30–43)	36 (30–43)	40 (36–46)	32 (28–41)	<0.001
Age in years, categorised					<0.001
< 30	124 (21.5 %)	86 (24.5 %)	13 (8.5 %)	25 (34.3 %)	
30–34	104 (18.0 %)	68 (19.4 %)	20 (13.1 %)	16 (21.9 %)	
35–39	120 (20.8 %)	71 (20.2 %)	41 (26.8 %)	8 (11.0 %)	
≥ 40	229 (39.7 %)	126 (35.9 %)	79 (51.6 %)	24 (32.9 %)	
Ethnic group^b^					0.181
Dutch	412 (73.8 %)	259 (75.5 %)	104 (70.8 %)	49 (72.1 %)	
Western, non-Dutch	72 (12.9 %)	44 (12.8 %)	23 (15.7 %)	5 (7.4 %)	
Non-western	74 (13.3 %)	40 (11.7 %)	20 (13.6 %)	14 (20.6 %)	
Sexual behaviour					
Median no. of male sex partners in preceding 6 months (IQR)	10 (5–20)	8 (5–15)	12 (6–30)	10 (5–20)	<0.001
No. of male sex partners in preceding 6 months					
1–4	119 (20.6 %)	83 (23.7 %)	22 (14.4 %)	14 (19.2 %)	<0.001
5–9	166 (28.8 %)	113 (32.2 %)	31 (20.3 %)	22 (30.1 %)	
10–24	188 (32.6 %)	112 (31.9 %)	53 (34.6 %)	23 (31.5 %)	
25–49	64 (11.1 %)	31 (8.8 %)	26 (17.0 %)	7 (9.6 %)	
≥ 50	40 (6.9 %)	12 (3.4 %)	21 (13.7 %)	7 (9.6 %)	

*Abbreviations*: *IQR* interquartile range

^a^Unless specified otherwise

^b^Ethnicity was missing for 8 HIV-negative participants, for 6 HIV-positive participants and for 5 HIV unaware participants

^c^P was calculated using *χ* ^2^-test for dichotomous and categorical variables and rank sum test for continuous variables

### Online and offline partner and partnership characteristics

Characteristics of online and offline partners and partnerships are shown in Table 2. The median age of the partners was 34 years (IQR 28–40). Compared to offline partners, more online partners were Dutch (61.3 % vs. 54.0 %; *P* < 0.001) and were defined as a known partner (77.7 % vs. 54.4 %; *P* < 0.001). The HIV status of online partners was more frequently reported as known (61.4 % vs. 49.4 %; *P* < 0.001), and in online partnerships, perceived HIV concordance was higher (49.0 % vs. 39.8 %; *P* < 0.001). Participants reported that their online partners more often knew the HIV status of the participant than offline partners (38.8 % vs. 27.2 %; *P* < 0.001). Participants more often reported multiple sexual contacts with online partners (50.9 % vs. 41.3 %; *P* < 0.001). Sex-related substance use, alcohol use, and group sex were less frequently reported with online partners.

**Table 2:** Characteristics of 1781 sexual partners and partnerships of 577 men who have sex with men, by means of how the participant established the partnership, Amsterdam, 2008-9

	Total	Online acquired partnerships	Offline acquired partnerships	*P* value^b^
	n(%)^e^	n(%)^e^	n(%)^e^	n(%)^e^
N	1781	878	903	
Demographics^a^				
Median age of partner in years (IQR)	34 (28–40)	34 (28–40)	34 (28–39)	0.546
Age of partner in years categorised				0.148
< 30	540 (30.5 %)	275 (31.5 %)	265 (29.6 %)	
30–34	371 (21.0 %)	174 (19.9 %)	197 (22.0 %)	
35–39	392 (22.2 %)	179 (20.5 %)	213 (23.8 %)	
≥ 40	466 (26.3 %)	246 (28.2 %)	220 (24.6 %)	
Median age difference in years participant - partner (IQR)^c^	4 (−2 to 10)	3 (−3 to 10)	4 (−2 to 10)	0.013
Age difference participant - partner, categorised				0.137
Partner >5 years older than participant	279 (15.8 %)	151 (17.3 %)	128 (14.3 %)	
Partner and participant differ ≤5 years	767 (43.4 %)	375 (42.9 %)	392 (43.8 %)	
Partner is >5 years younger than participant	723 (40.9 %)	348 (39.8 %)	375 (41.9 %)	
Ethnic group partner				<0.001
Dutch	974 (57.6 %)	520 (61.3 %)	454 (54.0 %)	
Western, non-Dutch	323 (19.1 %)	127 (15.0 %)	196 (23.3 %)	
Non-western	393 (23.3 %)	202 (23.8 %)	191 (22.7 %)	
Concordance between ethnic group of participant - partner				0.025
Concordant ethnicity	767 (43.1 %)	399 (45.4 %)	368 (40.8 %)	
Different ethnicity	1014 (56.9 %)	479 (54.6 %)	535 (59.3 %)	
Concordance in life styles participant - partner				0.353
No difference in life styles	703 (39.5 %)	338 (38.5 %)	365 (40.4 %)	
Differences in life styles	1078 (60.5 %)	540 (61.5 %)	538 (59.6 %)	
HIV status partner				
Perceived HIV status of partner				<0.001
HIV-negative	715 (40.2 %)	388 (44.2 %)	327 (36.2 %)	
HIV-positive	270 (15.2 %)	151 (17.2 %)	119 (13.2 %)	
Unaware	796 (44.7 %)	339 (38.6 %)	457 (50.6 %)	
Perceived HIV concordance participant - partner				<0.001
Same HIV status	789 (44.3 %)	430 (49.0 %)	359 (39.8 %)	
Different HIV status	135 (7.6 %)	72 (8.2 %)	63 (7.0 %)	
HIV status of participant or partner unknown	857 (48.1 %)	376 (42.8 %)	481 (53.3 %)	
Does partner know HIV status of participant?				<0.001
No	201 (11.3 %)	101 (11.5 %)	100 (11.1 %)	
Yes	585 (32.9 %)	340 (38.8 %)	245 (27.2 %)	
Possibly	993 (55.8 %)	436 (49.7 %)	557 (61.8 %)	
Sexual behaviour				
Partnership type				<0.001
Known partner	1137 (65.9 %)	661 (77.7 %)	476 (54.4 %)	
Anonymous partner	589 (34.1 %)	190 (22.3 %)	399 (45.6 %)	
Sex frequency with partner^d^				<0.001
Once	961 (54.0 %)	431 (49.1 %)	530 (58.7 %)	
2–5 times	624 (35.1 %)	344 (39.2 %)	280 (31.0 %)	
5–10 times	129 (7.3 %)	74 (8.4 %)	55 (6.1 %)	
> 10 times	66 (3.7 %)	28 (3.2 %)	38 (4.2 %)	
UAI				0.082
No	1339 (75.2 %)	646 (73.6 %)	693 (76.7 %)	
Yes	442 (24.8 %)	232 (26.4 %)	210 (23.3 %)	
Group sex with partner				0.003
No	1518 (85.3 %)	771 (87.9 %)	747 (82.7 %)	
Yes	262 (14.5 %)	106 (12.1 %)	156 (17.3 %)	
Paid sex with partner				0.055
No	1723 (96.9 %)	841 (96.0 %)	882 (97.7 %)	
Yes	56 (3.2 %)	35 (4.0 %)	21 (2.3 %)	
Sex-related substance use in partnership				
Any sex-related substance use				<0.001
No	633 (35.5 %)	344 (39.2 %)	289 (32.0 %)	
Yes	1148 (64.5 %)	534 (60.8 %)	614 (68.0 %)	
Sex-related alcohol use				<0.001
No	1143 (64.2 %)	627 (71.4 %)	516 (57.1 %)	
Yes	638 (35.8 %)	251 (28.6 %)	387 (42.9 %)	
Sex-related use of 2 or more substances other than alcohol				0.576
No	1467 (82.4 %)	719 (82.0 %)	748 (82.8 %)	
Yes	313 (17.6 %)	158 (18.0 %)	155 (17.2 %)	

*Abbreviations*: *UAI* unprotected anal intercourse, *IQR* interquartile range, *MSM* men who have sex with men

^a^Data were missing for partner’s age: online (4), offline (8); age difference: online (4), offline (8); partner’s ethnicity: online (29), offline (62); partnership type: online (27), offline (28); whether partner knows HIV status of participant: online (1), offline (1); sex frequency with partner: online (1); group sex: online (1); paid sex: online (2); use of 2 or more drugs during sex: online (1)

^b^P was calculated using logistic regression accounting for clustered observations

^c^Age difference was calculated by subtracting the age of the partner from the age of the participant

^d^The questionnaire stated overlapping categories

^e^Unless specified otherwise

In Additional file 1: Table S1 characteristics of partners and partnerships stratified by HIV status of participants are shown. UAI was much more common in partnerships of HIV-positive men (49 %) than in partnerships of HIV-negative men (13 %) or HIV-unaware men (28 %) (*P* < 0.001), but UAI was not significantly more common in online partnerships than in offline partnerships for any of the three HIV status groups. Table 3 shows the frequency of UAI by way of partner acquisition and the HIV status of the participant and the partner.

**Table 3:** Frequency of UAI by partner acquisition (online or offline) by participant’s and partner’s HIV status

			HIV status of participant								
			Negative			Positive			Unknown		
			No UAI	UAI	Total	No UAI	UAI	Total	No UAI	UAI	Total
HIV status of partner	Neg	Offline	228 (84.8 %)	41 (15.2 %)	269	32 (80.0 %)	8 (20.0 %)	40	15 (83.3 %)	3 (16.7 %)	18
		Online	267 (84.5 %)	49 (15.5 %)	316	33 (71.7 %)	13 (28.3 %)	46	21 (80.8 %)	5 (19.2 %)	26
		Total	495 (84.6 %)	90 (15.4 %)	585	65 (75.6 %)	21 (24.4 %)	86	36 (81.8 %)	8 (18.2 %)	44
	Pos	Offline	18 (78.3 %)	5 (21.7 %)	23	17 (18.9 %)	73 (81.1 %)	90	3 (50.0 %)	3 (50.0 %)	6
		Online	20 (76.9 %)	6 (23.1 %)	26	24 (21.1 %)	90 (79.0 %)	114	2 (18.2 %)	9 (81.8 %)	11
		Total	38 (77.6 %)	11 (22.5 %)	49	41 (20.1 %)	163 (79.9 %)	204	5 (29.4 %)	12 (70.6 %)	17
	Unk	Offline	234 (91.1 %)	23 (9.0 %)	257	83 (71.6 %)	33 (28.5 %)	116	63 (75.0 %)	21 (25.0 %)	84
		Online	171 (92.4 %)	14 (7.6 %)	185	63 (69.2 %)	28 (30.8 %)	91	45 (71.4 %)	18 (28.6 %)	63
		Total	405 (91.6 %)	37 (8.4 %)	442	146 (70.5 %)	61 (29.5 %)	207	108 (73.5 %)	39 (26.5 %)	147

*Abbreviations*: *UAI* unprotected anal intercourse, *Neg* negative HIV status, *Pos* positive HIV status, *Unk* HIV status unkown

### Association between online/offline dating and UAI

In univariate analysis, UAI was significantly more likely to occur in online than in offline partnerships (OR = 1.36 [95 % CI 1.03–1.81]) (Table 4). The self-perceived HIV status of the participant was strongly associated with UAI (OR = 11.70 [95 % CI 7.40–18.45]). The effect of dating location on UAI differed by HIV status, as can be seen best in Table 5. Table 5 shows the association of online dating using three different reference categories, one for each HIV status. Among HIV-positive men, UAI was more common in online compared to offline partnerships (OR = 1.61 [95 % CI 1.03–2.50]). Among HIV-negative men no association was apparent between UAI and online partnerships (OR = 1.07 [95 % CI 0.71–1.62]). Among HIV-unaware men, UAI was more common in online compared to offline partnerships, though not statistically significant (OR = 1.65 [95 % CI 0.79–3.44]).

**Table 4:** Univariate and multivariate associations between characteristics of the participant, the partner and partnership, and unprotected anal intercourse, in 1781 partnerships among 577 MSM. Results of random effects modelling. Amsterdam 2008-9

		Univariate	Model 1^a^	Model 2^b^	Model 3^c^
	No. of partnerships with UAI	OR (95 % CI)	aOR (95 % CI)	aOR (95 % CI)	aOR (95 % CI)
	n/N				
Dating location		*P* = 0.031			
Offline	210/903 (23.3 %)	1			
Online	232/878 (26.4 %)	1.36 (1.03–1.81)			
Self-perceived HIV status participant		*P* < 0.001			
HIV-negative	138/1076 (12.8 %)	1			
HIV-positive	245/497 (49.3 %)	11.70 (7.40–18.45)			
Unaware	59/208 (28.4 %)	3.55 (2.02–6.22)			
Dating location * HIV status participant		*P* < 0.001	*P* < 0.001	*P* < 0.001	*P* < 0.001
Offline partner * HIV negative participant	69/549 (12.6 %)	1	1	1	1
Offline partner * HIV positive participant	114/246 (46.3 %)	9.67 (5.60–16.71)	7.70 (4.43–13.39)	13.02 (7.05–24.03)	10.14 (5.32–19.30)
Offline partner * HIV unaware participant	27/108 (25.0 %)	2.88 (1.40–5.89)	2.63 (1.26–5.48)	7.02 (3.12–15.79)	4.30 (1.81–10.23)
Online partner * HIV negative participant	69/527 (13.1 %)	1.07 (0.71–1.62)	1.04 (0.69–1.59)	0.93 (0.60–1.43)	0.94 (0.59–1.48)
Online partner * HIV positive participant	131/251 (52.2 %)	15.55 (8.86–27.30)	12.67 (7.20–22.29)	18.65 (10.00–34.80)	16.41 (8.55–31.52)
Online partner * HIV unaware participant	32/100 (32.0 %)	4.73 (2.34–9.57)	4.87 (2.38–9.96)	12.99 (5.86–28.77)	11.00 (4.76–25.33)
Demographics of the participant					
Age in years categorised		*P* = 0.004	*P* = 0.219	*P* =0.230	*P* = 0.222
< 25	10/134 (7.5 %)	0.23 (0.08–0.70)	0.45 (0.16–1.22)	0.40 (0.14–1.12)	0.40 (0.13–1.19)
25–29	57/243 (23.5 %)	1.30 (0.61–2.75)	1.39 (0.70–2.78)	1.29 (0.63–2.64)	1.32 (0.62–2.82)
30–34	62/309 (20.1 %)	1	1	1	1
35–39	100/362 (27.6 %)	1.51 (0.77–2.98)	1.37 (0.74–2.55)	1.34 (0.71–2.55)	1.36 (0.69–2.66)
40–44	98/353 (27.8 %)	1.57 (0.79–3.12)	1.18 (0.63–2.22)	1.06 (0.55–2.05)	0.87 (0.43–1.75)
≥ 45	115/380 (30.3 %)	2.09 (1.06–4.11)	1.39 (0.75–2.59)	1.31 (0.67–2.57)	1.16 (0.57–2.35)
Ethnic group		*P* = 0.311	*P* = 0.151	*P* = 0.321	*P* = 0.470
Dutch	316/1285 (24.6 %)	1	1	1	1
Western, non-Dutch	45/221 (20.4 %)	0.89 (0.45–1.73)	0.71 (0.39–1.28)	0.76 (0.40–1.42)	0.68 (0.35–1.31)
Non-western	67/221 (30.3 %)	1.60 (0.83–3.06)	1.49 (0.85–2.63)	1.37 (0.74–2.53)	1.05 (0.55–2.01)
Sexual behaviour of the participant					
No. of male sex partners in preceding 6 months, categorized		*P* < 0.001	*P* = 0.255	*P* = 0.345	*P* = 0.570
< 5	62/333 (18.6 %)	1	1	1	1
5–9	107/528 (20.3 %)	1.34 (0.74–2.43)	1.16 (0.67–2.00)	1.22 (0.69–2.14)	1.11 (0.62–2.01)
10–24	149/574 (26.0 %)	1.79 (0.99–3.22)	1.27 (0.75–2.18)	1.27 (0.73–.2.20)	1.09 (0.61–1.95)
25–49	63/211 (30.0 %)	2.27 (1.08–4.76)	1.40 (0.71–2.78)	1.36 (0.67–2.77)	1.18 (0.56–2.48)
≥ 50	61/135 (45.2 %)	6.79 (2.86–16.13)	2.48 (1.12–5.50)	2.43 (1.06–5.56)	2.08 (0.86–5.00)
Characteristics of the partnership					
Age difference (yrs) participant - partner, categorised		*P* = 0.482		*P* = 0.121	*P* = 0.109
Partner is >5 years older than participant	72/279 (25.8 %)	1		1	1
Partner & participant differ ≤5 years in age	180/767 (23.5 %)	0.74 (0.45–1.21)		0.63 (0.37–1.07)	0.68 (0.39–1.19)
Partner is >5 years younger than participant	187/723 (25.9 %)	0.79 (0.46–1.35)		0.85 (0.47–1.56)	1.01 (0.53–1.92)
Etnic concordance participant - partner		*P* = 0.60		*P* = 0.609	*P* = 0.320
Concordant ethnicity	193/767 (25.2 %)	1		1	1
Different ethnicity	249/1014 (24.6 %)	0.96 (0.68–1.34)		1.10 (0.76–1.60)	1.22 (0.82–1.81)
Concordance in life styles participant - partner		*P* = 0.115		*P* = 0.023	*P* = 0.015
No difference in life styles	186/703 (26.5 %)	1		1	1
Differences in life styles	256/1078 (23.8 %)	0.75 (0.52–1.07)		0.66 (0.46–0.95)	0.63 (0.43–0.92)
HIV concordance participant -partner		*P* <0.001		*P* < 0.001	*P* < 0.001
Same HIV status	253/789 (32.1 %)	1		1	1
Different HIV status	32/135 (23.7 %)	0.24 (0.12–0.47)		0.14 (0.07–0.27)	0.15 (0.08–0.30)
HIV status unknown of participant or partner	157/857 (18.3 %)	0.29 (0.20–0.43)		0.19 (0.12–0.29)	0.25 (0.16–0.40)
Partnership type		*P* = 0.055			*P* = 0.413
Known partner	289/1137 (25.4 %)	1			1
Anonymous partner	135/589 (22.9 %)	0.70 (0.49–1.01)			1.20 (0.77–1.88)
Sex frequency with partner^d^		*P* < 0.001			*P* < 0.001
Once	186/961 (19.4 %)	1			1
2–5 times	167/624 (26.8 %)	1.98 (1.40–2.80)			1.74 (1.17–2.58)
5–10 times	50/129 (38.8 %)	3.58 (1.99–6.45)			2.10 (1.09–4.08)
> 10 times	39/66 (59.1 %)	16.29 (7.07–37.52)			10.38 (4.29–25.15)
Group sex with partner		*P* < 0.001			*P* = 0.077
No	337/1518 (22.2 %)	1			1
Yes	105/262 (40.1 %)	2.54 (1.65–3.89)			1.55 (0.95–2.52)
Sex-related substance use in partnership					
Sex-related use of at least 2 substances other than alcohol		*P* < 0.001			*P* < 0.001
No	288/1467 (19.6 %)	1			1
Yes	154/313 (49.2 %)	5.37 (3.51–8.21)			2.32 (1.44–3.75)

*Abbreviations*: *UAI* Unprotected anal intercourse, *MSM* men who have sex with men, *OR* odds ratio, *aOR* adjusted odds ratio

^a^Model 1 includes, besides dating location, the following co-variates of the participant: age; self-perceived HIV status; and no. of sex partners in preceding 6 months

^b^Model 2 additionally includes variables concerning the partnership

^c^Model 3 additionally includes variables concerning sexual behavior in the partnership

^d^Overlapping categories were stated in the questionnaire

^*^indicates interaction term

**Table 5:** The association between online dating and UAI, with three different reference categories, in 1781 partnerships among 577 MSM. Results of multivariable random effects modelling. Amsterdam 2008-9

		Univariate	Model 1	Model 2	Model 3
	No. of partnerships with UAI	OR (95 % CI)	aOR (95 % CI)	aOR (95 % CI)	aOR (95 % CI)
	n/N (%)				
Dating location * HIV status participant		*P* = 0.754	*P* = 0.843	*P* = 0.727	*P* = 0.778
Offline partner * HIV negative participant	69/549 (12.6 %)	1	1	1	1
Online partner * HIV negative participant	69/527 (13.1 %)	1.07 (0.71–1.62)	1.04 (0.69–1.59)	0.93 (0.60–1.43)	0.94 (0.59–1.48)
		*P* = 0.035	*P* = 0.029	*P* = 0.140	*P* = 0.069
Offline partner * HIV positive participant	114/246 (46.3 %)	1	1	1	1
Online partner * HIV positive participant	131/251 (52.2 %)	1.61 (1.03–2.50)	1.65 (1.05–2.57)	1.43 (0.89–2.31)	1.62 (0.96–2.72)
		*P* = 0.187	*P* = 0.113	*P* = 0.118	*P* = 0.027
Offline partner * HIV unaware participant	27/108 (25.0 %)	1	1	1	1
Online partner * HIV unaware participant	32/100 (32.0 %)	1.65 (0.79–3.44)	1.85 (0.86–3.98)	1.85 (0.86–4.00)	2.55 (1.11–5.86)

*Abbreviations*: *UAI* unprotected anal intercourse, *MSM* men who have sex with men, *OR* odds ratio, *aOR* adjusted odds ratio

^*^indicates interaction term

In the first multivariate model (Tables 4 and 5), including only demographic and sexual behaviour variables of the participant, the associations between online dating and UAI were very similar to those in the univariate model (aOR = 1.65 [95 % CI 1.05–2.57] for HIV-positive men, and aOR = 1.04 [95 % CI 0.69–1.59] for HIV-negative men, and aOR = 1.85 [95 % CI 0.86–3.98] for HIV-unaware men) (Table 5).

In multivariate model 2 (Tables 4 and 5), variables concerning the partnership were added (lifestyle concordance, ethnic concordance, and HIV concordance). Among HIV-positive men the effect of meeting location on UAI was smaller and no longer significant (aOR = 1.43 [95 % CI 0.89–2.31]; Table 5).

In multivariate model 3 (Tables 4 and 5), additionally including variables concerning sexual behaviour in the partnership (sex-related multiple drug use, sex frequency and partner type), the independent effect of online dating location on UAI became somewhat stronger (though not significant) for the HIV-positive men (aOR = 1.62 [95 % CI; 0.96–2.72]), but remained similar for HIV-negative men (aOR = 0.94 [95 % CI 0.59–1.48]). The effect of online dating on UAI became stronger (and significant) for HIV-unaware men (aOR = 2.55 [95 % CI 1.11–5.86]) (Table 5).

Perceived concordance of HIV status was associated with UAI in models 2 and 3 (Table 4). In model 3, HIV discordance (aOR = 0.15 [95 % CI 0.08–0.30]) or unknown HIV concordance (aOR = 0.25 [95 % CI 0.16–0.40]) were negatively associated with UAI (Table 4).

We investigated the effect of self-perceived HIV concordance on UAI separately for HIV-positive and HIV-negative men. The effect of self-perceived HIV concordance on UAI was very strong in HIV-positive men (aOR 24.09 [95 % CI 9.17–63.31]), but not in HIV-negative men (aOR 0.42 [95 % CI 0.14–1.27]).

The number of sex partners in the preceding 6 months of the index was also associated with UAI (OR = 6.79 [95 % CI 2.86–16.13] for those with 50 or more recent sex partners compared to those with fewer than 5 recent sex partners). UAI was significantly more likely if more sex acts had occurred in the partnership (OR = 16.29 [95 % CI 7.07–37.52] for >10 sex acts within the partnership compared to only one sex act). Other factors significantly associated with UAI were group sex within the partnership, and sex-related multiple drug use within partnership.

When we repeated the analyses using a different categorization of self-perceived HIV status (assigning those who indicated “I think I may be HIV positive” to the category Unknown, rather than to the category HIV-positive), the results were unchanged. A sensitivity analysis, including only data of partnerships in which only one sex act had occurred, showed similar results regarding the association between online dating and UAI (data not shown).

## Discussion

In this large study among MSM attending the STI clinic in Amsterdam, we found no evidence that online dating was independently associated with a higher risk of UAI than offline dating. For HIV-negative men this lack of assocation was clear (aOR = 0.94 [95 % CI 0.59–1.48]); among HIV-positive men there was a non-significant association between online dating and UAI (aOR = 1.62 [95 % CI 0.96–2.72]). Only among men who indicated they were not aware of their HIV status (a small group in this study), UAI was more common with online than offline partners.

Among HIV-positive men, in univariate analysis UAI was reported significantly more often with online partners than with offline partners. When adjusting for partner characteristics, the effect of online/offline dating on UAI among HIV-positive MSM became somewhat smaller and became non-significant; this suggests that differences in partnership factors between online and offline partnerships are responsible for the increased UAI in online established partnerships. This might be due to a mediating effect of more information on partners, (including perceived HIV status) on UAI, or to other factors. Among HIV-negative men no effect of online dating on UAI was observed, either in univariate or in any of the multivariate models. Among HIV-unaware men, online dating was associated with UAI but only significant when adding partner and partnership variables to the model.

The data also suggest that concordance in HIV status is an important predictor for UAI in all groups (HIV- positive, -negative, and –unaware men). Concordance in HIV status may be more important for HIV-positive men than for others, and perhaps Internet dating helps to assess each other’s HIV status more easily.

A key strength of this study was that it explored the relation between online dating and UAI among MSM who had recent sexual contact with both online and offline casual partners. This avoided bias caused by potential differences between men only dating online and those only dating offline, a weakness of several previous studies. By recruiting participants at the largest STI outpatient clinic in the Netherlands we could include a large number of MSM, and avoid potential differences in men sampled through Internet or face-to-face interviewing, weaknesses in some previous studies [3, 11].

This study had some limitations. MSM visiting the STI outpatient clinic did so with a reason; they probably had higher sexual risk behaviour than the general MSM population. Therefore extrapolation of our results to the general MSM population might not be warranted.

The probability of UAI increased with the number of sex acts in a partnership. Unfortunately, information on UAI during the first sex act was lacking. A sensitivity analysis, using data of partnerships with one sex act only showed similar results and did not lead to different conclusions.

We observed that, when dating online, both HIV-positive and HIV-negative men more often had a perception (whether correct or not, we don’t know) of the partner’s HIV status compared to when they date offline.

Perceived concordance in HIV status was higher with online than with offline partners for HIV-positive as well as HIV-negative men. Data also suggests that men thought that their online partners knew their HIV status more often than their offline partners. This could reflect the online opportunities in anonymously exchanging information on HIV status prior to sexual contact, but at the same time also reflects the existing barriers MSM experience with face-to-face disclosure [19, 20].

In general, HIV-positive men report more UAI than HIV-negative men. Stratified analyses examining UAI and HIV concordance showed that HIV-positive men appear to engage in UAI especially with other HIV-positive men; UAI with discordant and HIV status unknown partners was significantly less common. For HIV-negative men, UAI was reported relatively infrequently and appeared not strongly related to the HIV status of the partner.

Online dating was not associated with UAI among HIV-negative men, a finding in agreement with some previous studies, mostly among young men [21], but in contrast with other studies [1–5]. This may be due to the fact that most earlier studies compared sexual behaviour of two groups of MSM rather than comparing two sexual behaviour patterns within one group of men. However it may also reflect secular changes; perhaps in the beginning of online dating a more high-risk group of men used the Internet, and over time online dating normalized and less high-risk MSM nowadays also use the Internet for dating.

For HIV-unaware men the effect of dating location on UAI did not change by adding partner characteristics, but it increased when adding lifestyle and drug use. It is hard to assess the actual risk for HIV for these men: do they behave as HIV-negative men who are trying to protect themselves from HIV infection, or as HIV-positive men trying to protect their HIV-negative partner from HIV infection? A study by Horvath et al. reported that 72 % of men who were never tested for HIV, profiled themselves online as being HIV-negative, which might be problematic if they are HIV-positive and engage in UAI with HIV-negative partners [12]. Previously Matser et al. reported that 1.7 % of the unaware and perceived HIV-negative MSM were tested HIV-positive. The study population included the MSM reported in this study [15].

The results strongly suggest serosorting behaviour among HIV-positive men (i.e., establishing that partners are HIV concordant prior to practicing UAI), but less so in HIV-negative men. As we did not ask whether the participant intentionally explored the HIV status of his partner in order to engage in UAI, we cannot define UAI with concordant partners as serosorting.

Because decisions on UAI appear to be partly based on perceived HIV concordance, accurate knowledge of one’s own and the partner’s HIV status is important. In HIV-negative men and HIV status-unaware men, decisions on UAI will not only be based on perceived HIV status of the partner but also on one’s own negative status. HIV serosorting is challenged by the frequency of HIV testing and the HIV window phase during which individuals can transmit HIV but cannot be diagnosed with the commonly used HIV tests. Therefore serosorting cannot be regarded as a very effective method of avoiding HIV transmission [22]. Besides interventions to stimulate the uptake of HIV and STI testing in sexually active men, interventions to caution against UAI based on perceived HIV-negative concordant status are in order, irrespective of whether this concerns online or offline dating.

Dating online may offer other opportunities for communication on HIV status than dating in physical environments. Facilitating more online HIV status disclosure during partner seeking makes serosorting easier. However, serosorting may increase the burden of other STI and will not prevent HIV infection entirely. Interventions to prevent HIV transmission should especially be directed at HIV-negative and unaware MSM and stimulate timely HIV testing (i.e., after risk events or when experiencing symptoms of seroconversion illness) as well as regular testing when sexually active.

New research should stay up-to-date when it comes to rapid changing dating methods and sero-adaptive behaviours (like viral sorting and pre exposure prophylaxis). With every new way of dating and preventive opportunities, the rules of engagements will vary. Our data are 8 years old and internet-based dating has developed since then. Nevertheless these results are useful, as they show how internet-based partner acquisition may lead to more information on the sex partner, and this may impact on the frequency of UAI.

## Conclusions

HIV-positive MSM are more likely to practise UAI with partners dated online than with partners dated offline; however after correction for partner and partnership characteristics, online partnership acquisition was not associated with a significantly increased risk of UAI.

## Abbreviations

AMC, Academic Medical Center; aOR, adjusted odds ratio; CI, confidence interval; CINIMA, Center for Infection and Immunology Amsterdam; DAG, directed acyclic graph; HIV, human immuno-deficiency virus; i.e., id est, it is, for example; IQR, interquartile range; MEC, Medical Ethics Committee; MSM, men who have sex with men; OR, odds ratio; RIVM, National Institute of Public Health and the Environment, Centre for Infectious Disease Control; STI, sexually transmitted infection; UAI, unprotected anal intercourse; UMCU, University Medical Center Utrecht

## Additional file

Additional file 1: Table S1. Characteristics of 1781 sexual partnerships of 577 men who have sex with men, by means how the participant established the partnership, and stratified by HIV status; 1076 partnerships of 351 HIV negative men, 497 partnerships of 153 HIV positive men, 208 partnerships of 73 HIV status unaware men, Amsterdam, 2008-9. (DOCX 27 kb)
