# Supplementary material for: Does online dating lead to higher sexual risk behaviour? A cross-sectional study among MSM in Amsterdam, the Netherlands
Source: BMC Infect Dis. 2016 Jun 14;16:288. doi: 10.1186/s12879-016-1637-5 (PMC4907249; doi:10.1186/s12879-016-1637-5)
Supplement: Additional file 1: Table S1. — Characteristics of 1781 sexual partnerships of 577 men who have sex with men, by means how the participant established the partnership, and stratified by HIV status; 1076 partnerships of 351 HIV negative men, 497 partnerships of 153 HIV positive men, 208 partnerships of 73 HIV status unaware men, Amsterdam, 2008-9. (DOCX 27 kb) [file 12879_2016_1637_MOESM1_ESM.docx]

**Supplementary Table 1. Characteristics of 1,781 sexual partnerships of 577 men who have sex with men, by means how the participant established the partnership, and stratified by HIV status; 1,076 partnerships of 351 HIV negative men, 497 partnerships of 153 HIV positive men, 208 partnerships of 73 HIV status unaware men, Amsterdam, 2008-9.**

|  | **Participant = HIV negative** | |  | **Participant = HIV positive** | |  |  | **Participant = HIV unaware** | | |  |
| --- | --- | --- | --- | --- | --- | --- | --- | --- | --- | --- | --- |
|  | Online acquired partnerships | Offline acquired partnerships | **P value^b^** | Online acquired partnerships | Offline acquired partnerships | **P value^b^** | Online acquired partnerships | | Offline acquired partnerships | | **P value^b^** |
|  | n=527 | n=549 |  | n=251 | n=246 |  | n=100 | | | n=108 |  |
| **Demographics^a^** |  |  |  |  |  |  |  | | |  |  |
| Median age of partner in years (IQR) | 32 (26-39) | 32 (28-38) | 0.585 | 36 (30-43) | 35 (30-40) | 0.240 | 32 (25.5 to 40) | | | 31 (25 to 37) | 0.416 |
| Age of partner in years categorised |  |  | 0.376 |  |  | 0.636 |  | | |  | 0.214 |
| < 30 | 188 (36.0%) | 179 (32.8%) |  | 47 (18.7%) | 42 (17.3%) |  | 40 (40.0%) | | | 44 (41.1%) |  |
| 30-34 | 117 (22.4%) | 132 (24.2%) |  | 40 (15.9%) | 48 (19.8%) |  | 17 (17.0%) | | | 17 (15.9%) |  |
| 35-39 | 95 (18.2%) | 118 (21.7%) |  | 67 (26.7%) | 66 (27.2%) |  | 17 (17.0%) | | | 29 (27.1%) |  |
| *≥*40 | 123 (23.5%) | 116 (21.3%) |  | 97 (38.7%) | 87 (35.8%) |  | 26 (26.0%) | | | 17 (15.9%) |  |
|  |  |  |  |  |  |  |  | | |  |  |
| Median age difference in years participant - partner (IQR)^c^ | 3 (-3 to 10) | 3 (-3 to 10) | 0.571 | 3 (-3 to 10) | 5 (0 to 11) | 0.022 | 2 (-6.5 to 8) | | | 2 (-4 to 7) | 0.574 |
| Age difference in years participant - partner, categorised |  |  | 0.756 |  |  | 0.045 |  | | |  | 0.420 |
| Partner >5 yrs older than participant | 87 (16.6%) | 84 (15.4%) |  | 38 (15.1%) | 24 (9.9%) |  | 26 (26.0%) | | | 20 (18.7%) |  |
| Partner and participant differ ≤5 yrs | 218 (41.7%) | 237 (43.5%) |  | 119 (47.4%) | 108 (44.4%) |  | 38 (38.0%) | | | 47 (43.9%) |  |
| Partner is >5 yrs younger than participant | 218 (41.7%) | 224 (41.1%). |  | 94 (37.5%) | 111 (45.7%) |  | 36 (36.0%) | | | 40 (37.4%) |  |
| Ethnic group partner |  |  | <0.001 |  |  | 0.017 |  | | |  | 0.066 |
| Dutch | 298 (57.9%) | 279 (53.9%) |  | 163 (67.9%) | 124 (55.6%) |  | 59 (62.8%) | | | 51 (51.0%) |  |
| Western, non-Dutch | 75 (14.6%) | 122 (23.6%) |  | 41 (17.1%) | 50 (22.4%) |  | 11 (11.7%) | | | 24 (24.0%) |  |
| Non-western | 142 (27.6%) | 117 (22.6%) |  | 36 (15.0%) | 49 (22.0%) |  | 24 (25.5%) | | | 25 (25.0%) |  |
| Concordance between ethnic group of participant and partner |  |  | 0.354 |  |  | 0.041 |  | | |  | 0.210 |
| Concordant ethnicity | 233 (44.2%) | 229 (41.7%) |  | 120 (47.8%) | 97 (39.4%) |  | 46 (46.0%) | | | 42 (38.9%) |  |
| Different ethnicity | 294 (55.8%) | 320 (58.3%) |  | 131 (52.2%) | 149 (60.6%) |  | 54 (54.0%) | | | 66 (61.1%) |  |
| Concordance in life styles participant - partner |  |  | 0.828 |  |  | 0.624 |  | | |  | 0.189 |
| No difference in life styles | 211 (40.0%) | 223 (40.6%) |  | 83 (33.1%) | 86 (35.0%) |  | 44 (44.0%) | | | 56 (51.9%) |  |
| Differences in life styles | 316 (60.0%) | 326 (59.4%) |  | 168 (66.9%) | 160 (65.0%) |  | 56 (56.0%) | | | 52 (48.2%) |  |
|  |  |  |  |  |  |  |  | | |  |  |
| **HIV status** |  |  |  |  |  |  |  | | |  |  |
| Perceived HIV status of partner |  |  | <0.001 |  |  | 0.015 |  | | |  | 0.031 |
| HIV-negative | 316 (60.0%) | 269 (49.0%) |  | 46 (18.3%) | 40 (16.3%) |  | 26 (26.0%) | | | 18 (16.7%) |  |
| HIV-positive | 26 (5.0%) | 23(4.2%) |  | 114 (45.4%) | 90 (36.6%) |  | 11 (11.0%) | | | 6 (5.6%) |  |
| Unaware | 185 (35.1%) | 257(46.8%) |  | 91 (36.3%) | 116 (47.2%) |  | 63 (63.0%) | | | 84 (77.8%) |  |
| Concordance in HIV status participant - partner |  |  | <0.001 |  |  | 0.015 |  | | |  | -- |
| Same HIV status | 316 (60.0%) | 269 (49.0%) |  | 114 (45.4%) | 90 (36.6%) |  | -- | | | -- |  |
| Different HIV status | 26 (5.0%) | 23(4.2%) |  | 46 (18.3%) | 40 (16.3%) |  | -- | | | -- |  |
| HIV status of participant or partner unknown | 185 (35.1%) | 257(46.8%) |  | 91 (36.3%) | 116 (47.2%) |  | -- | | | -- |  |
| Does partner know HIV status of participant? |  |  | <0.001 |  |  | 0.001 |  | | |  | 0.016 |
| No | 61 (11.6%) | 68 (12.4%) |  | 31 (12.4%) | 26 (10.6%) |  | 9 (9.0%) | | | 6 (5.6%) |  |
| Yes | 186 (35.4%) | 135 (24.6%) |  | 129 (51.4%) | 95 (38.6%) |  | 25 (25.0%) | | | 15 (13.9%) |  |
| Possibly | 279 (53.0%) | 345 (63%) |  | 91 (36.3%) | 125 (50.8%) |  | 66 (66.0%) | | | 87 (80.6%) |  |
|  |  |  |  |  |  |  |  | | |  |  |
| **Sexual Behaviour** |  |  |  |  |  |  |  | | |  |  |
| Partnership type |  |  | <0.001 |  |  | <0.001 |  | | |  | 0.207 |
| Known partner | 408 (79.8%) | 305 (57.1%) |  | 179 (73.7%) | 100 (42.2%) |  | 74 (76.3%) | | | 71 (68.3%) |  |
| Anonymous partner | 103 (20.2%) | 229 (42.9%) |  | 64 (26.3%) | 137 (57.8%) |  | 23 (23.7%) | | | 33 (31.7%) |  |
| Sex frequency with partner^d^ |  |  | 0.033 |  |  | 0.013 |  | | |  | 0.051 |
| Once | 273 (51.9%) | 333 (60.7%) |  | 115 (45.8%) | 140 (56.9%) |  | 43 (43.0%) | | | 57 (52.8%) |  |
| 2-5 times | 203 (38.6%) | 173 (31.5%) |  | 95 (37.9%) | 77 (31.3%) |  | 46 (46.0%) | | | 30 (27.8%) |  |
| 5-10 times | 35 (6.7%) | 27 (4.9%) |  | 34 (13.6%) | 16 (6.5%) |  | 5 (5.0%) | | | 12 (11.1%) |  |
| >10 times | 15 (2.9%) | 16 (2.9%) |  | 7 (2.8%) | 13 (5.3%) |  | 6 (6.0%) | | | 9 (8.3%) |  |
| UAI |  |  | 0.788 |  |  | 0.15 |  | | |  | 0.187 |
| No | 458 (87.0%) | 480 (87.4%) |  | 120 (47.8%) | 132 (53.7%) |  | 68 (68.0%) | | | 81 (75.0%) |  |
| Yes | 69 (13.1%) | 69 (12.6%) |  | 131 (52.2%) | 114 (46.3%) |  | 32 (32.0%) | | | 27 (25.0%) |  |
| Group sex with partner |  |  | 0.002 |  |  | 0.393 |  | | |  | 0.104 |
| No | 487 (92.6%) | 479 (87.3%) |  | 196 (78.1%) | 183 (74.4%) |  | 88 (88.0%) | | | 85 (78.7%) |  |
| Yes | 39 (7.4%) | 70 (12.8%) |  | 55 (21.9%) | 63 (25.6%) |  | 12 (12.0%) | | | 23 (21.3%) |  |
| Paid sex with partner |  |  | 0.110 |  |  | 0.854 |  | | |  | <0.001 |
| No | 504 (96.0%) | 537 (97.8%) |  | 243 (96.8%) | 239 (97.2%) |  | 94 (94.0%) | | | 106 (98.2%) |  |
| Yes | 21 (4.0%) | 12 (2.2%) |  | 8 (3.2%) | 7 (2.9%) |  | 6 (6.0%) | | | 2 (1.9%) |  |
|  |  |  |  |  |  |  |  | | |  |  |
| **Sex-related substance use in partnership** |  |  |  |  |  |  |  | | |  |  |
| Any sex-related substance use |  |  | 0.001 |  |  | 0.381 |  | | |  | 0.254 |
| No | 234 (44.4%) | 192 (35.0%) |  | 65 (25.9%) | 56 (22.8%) |  | 45 (45.0%) | | | 41 (38.0%) |  |
| Yes | 293 (55.6%) | 357 (65.0%) |  | 186 (74.1%) | 190 (77.2%) |  | 55 (55.0%) | | | 67 (62.0%) |  |
| Sex-related alcohol use |  |  | <0.001 |  |  | 0.006 |  | | |  | 0.042 |
| No | 363 (68.9%) | 288 (52.5%) |  | 197 (78.5%) | 169 (68.7%) |  | 67 (67.0%) | | | 59 (54.6%) |  |
| Yes | 164 (31.1%) | 261 (47.5%) |  | 54 (21.5%) | 77 (31.3%) |  | 33 (33.0%) | | | 49 (45.4%) |  |
| Sex-related use of 2 or more substances other than alcohol |  |  | 0.380 |  |  | 0.456 |  | | |  | 0.513 |
| No | 473 (89.9%) | 502 (91.4%) |  | 168 (66.9%) | 158 (64.2%) |  | 78 (78.0%) | | | 88 (81.5%) |  |
| Yes | 53 (10.1%) | 47 (8.6%) |  | 83 (33.1%) | 88 (35.8%) |  | 22 (22.0%) | | | 20 (18.5%) |  |

a Data were missing for partner’s age and age difference; HIV neg (8), HIV pos (3), HIV unaware (1), partner’s ethnicity HIV neg (43), HIV pos (34), HIV unaware (14), partnership type; HIV neg (31), HIV pos (17), HIV unaware (12), whether partner knows HIV status of the participant HIV neg (2), sex frequency with partner; HIV neg (1), group sex; HIV negative (1), paid sex; HIV negative (2), use of 2 or more drugs during sex; HIV negative (1).

b. P was calculated using logistic regression accounting for clustered observations.

c Age difference was calculated by subtracting the age of the partner from the age of the participant.

d The questionnaire stated overlapping categories.
Abbreviations: UAI Unprotected anal intercourse; IQR interquartile range; MSM men who have sex with men.
